# An Ensemble Learning Method for Emotion Charting Using Multimodal Physiological Signals

**DOI:** 10.3390/s22239480

**Published:** 2022-12-04

**Authors:** Amna Waheed Awan, Syed Muhammad Usman, Shehzad Khalid, Aamir Anwar, Roobaea Alroobaea, Saddam Hussain, Jasem Almotiri, Syed Sajid Ullah, Muhammad Usman Akram

**Affiliations:** 1 Department of Computer Engineering, Bahria University, Islamabad 44000, Pakistan; 2 Department of Creative Technologies, Faculty of Computing and AI, Air University, Islamabad 44000, Pakistan; 3 School of Computing and Engineering, The University of West London, London W5 5RF, UK; 4 Department of Computer Science, College of Computers and Information Technology, Taif University, P.O. Box 11099, Taif 21944, Saudi Arabia; 5 School of Digital Science, Universiti Brunei Darussalam, Jalan Tungku Link, Gadong BE1410, Brunei; 6 Department of Information and Communication Technology, University of Agder (UiA), N-4898 Grimstad, Norway; 7 Department of Electrical and Computer Engineering, Villanova University, Villanova, PA 19085, USA; 8 College of Eletrical and Mechanical Engineering (E & ME), National University of Science and Technology (NUST), Islamabad 44000, Pakistan

**Keywords:** emotion charting, EEG signals, physiological signals, ECG signals, ICA, stacked autoencoder, ensemble classifier

## Abstract

Emotion charting using multimodal signals has gained great demand for stroke-affected patients, for psychiatrists while examining patients, and for neuromarketing applications. Multimodal signals for emotion charting include electrocardiogram (ECG) signals, electroencephalogram (EEG) signals, and galvanic skin response (GSR) signals. EEG, ECG, and GSR are also known as physiological signals, which can be used for identification of human emotions. Due to the unbiased nature of physiological signals, this field has become a great motivation in recent research as physiological signals are generated autonomously from human central nervous system. Researchers have developed multiple methods for the classification of these signals for emotion detection. However, due to the non-linear nature of these signals and the inclusion of noise, while recording, accurate classification of physiological signals is a challenge for emotion charting. Valence and arousal are two important states for emotion detection; therefore, this paper presents a novel ensemble learning method based on deep learning for the classification of four different emotional states including high valence and high arousal (HVHA), low valence and low arousal (LVLA), high valence and low arousal (HVLA) and low valence high arousal (LVHA). In the proposed method, multimodal signals (EEG, ECG, and GSR) are preprocessed using bandpass filtering and independent components analysis (ICA) for noise removal in EEG signals followed by discrete wavelet transform for time domain to frequency domain conversion. Discrete wavelet transform results in spectrograms of the physiological signal and then features are extracted using stacked autoencoders from those spectrograms. A feature vector is obtained from the bottleneck layer of the autoencoder and is fed to three classifiers SVM (support vector machine), RF (random forest), and LSTM (long short-term memory) followed by majority voting as ensemble classification. The proposed system is trained and tested on the AMIGOS dataset with *k*-fold cross-validation. The proposed system obtained the highest accuracy of 94.5% and shows improved results of the proposed method compared with other state-of-the-art methods.

## 1. Introduction

**Figure 1:**
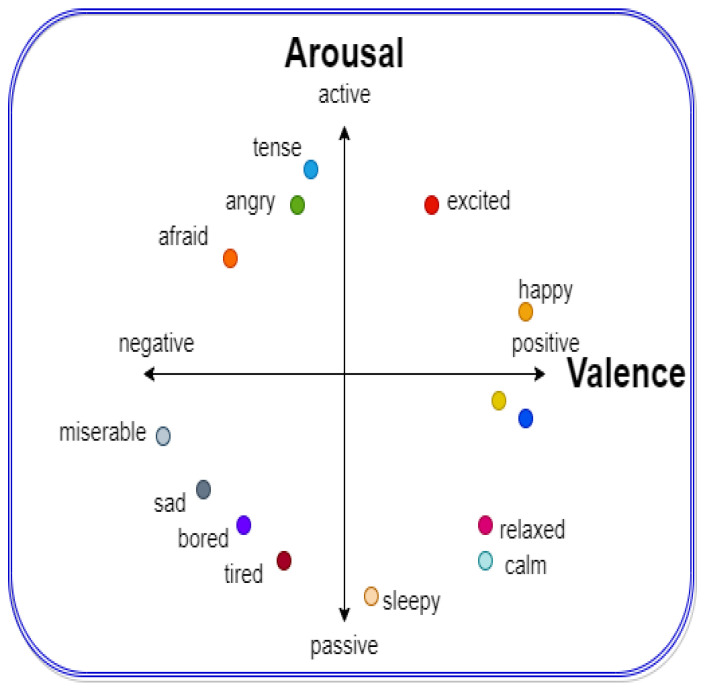
Valence arousal model [7].

Human emotion is the complex feelings that result in physiological as well as psychological changes [1,2]. These changes force us to respond to certain stimuli and make changes in our thoughts and behavior. Human emotions are recognized using physiological and non-physiological signals [3]. Emotions are usually described as valence and arousal. Valence describes positive and negative emotions and arousal describes the strength of excitement [1]. In the literature, most of the methods of emotion recognition based on physiological signals classify valence and arousal into low and high levels. However, many researchers performed emotions classification using four categories, i.e., high valence and high arousal (HVHA), low valence and low arousal (LVLA), high valence and low arousal (HVLA), and low valence high arousal (LVHA) as depicted in Figure 1.

A lot of effort has been put into designing an intelligent emotion recognition system using both physiological and non-physiological signals. Electroencephalogram (EEG), electrocardiogram (ECG), galvanic skin response (GSR), and blood volume pulse (BVP) are popular physiological signals while facial expressions, speech, and body gestures are non-physiological signals [3]. Physiological signals are more effective for emotion recognition as they are captured directly from the human body and cannot be manipulated so they give a true perception of human intuitions. Therefore, emotion recognition using physiological signals has become a hot topic in research because these signals represent the internal emotional state of a human and they cannot be masked intentionally. Emotion recognition has a wide range of applications such as physiological healthcare monitoring especially human’s mental health [4,5], general security purposes [6], and various bio-inspired human–machine interfaces, etc. [7].

Emotion recognition using physiological signals has gained much attention in recent research and many of the physiological signals such as electrocardiogram (ECG), galvanic skin response (GSR), electroencephalogram (EEG), and respiratory suspended particulate (RSP) have been effectively used for emotion recognition. All these physiological signals are captured and measured by body sensors and are more effective means for computing emotional responses. A lot of struggles have been done in the literature to build a critical relationship between the changes invoked by emotions and what impact they leave on physiological signals. In particular, EEG and ECG exhibit strong associations between their waveform and emotional characteristics [8]. Furthermore, the acquisition process for ECG and EEG is inexpensive, non-invasive, and fast, which makes them suitable tools for emotions recognition [9]. Many emotion recognition methods have been proposed in the literature using EEG and ECG signals [3,10,11], where multimodal techniques give the highest recognition precision than systems with a single modality. Topic et al. extracted feature maps from EEG signals of four different datasets, i.e., DREAMER, AMIGOS, SEED, and DEAP. Garg et al. came up with an overlapping sliding window (OSW) modeling framework for emotion recognition using EEG signals of the AMIGOS dataset. While Shukla et al. extracted EDA features from EEG signals of AMIGOS datasets and used three different feature selection techniques for emotion recognition. However, on the other side, many models have used the fusion of GSR and ECG signals such as [1,7,12]. Rahim et al. used shimmer sensors for extracting ECG and GSR signals from the human body and Granados et al. performed emotion detection using ECG and GSR signals of the AMIGOS dataset. Similarly, Dar et al. classified emotions into four categories, i.e., HAHV, LALV, LAHV, and HALV using GSR and ECG signals of AMIGOS and DREAMER datasets for emotion detection. 

Conventionally, emotion recognition is performed in three main steps, i.e., preprocessing, feature extraction, and classification [13,14,15]. Preprocessing involves signal filtering and noise removal which was addressed in many works such as [7,12], however, baseline removal was completed along with basic preprocessing in [15]. Similarly, it is very important to extract discriminant features from physiological signals to perform emotion recognition in an efficient way. Garg et al. [3] extracted Fourier and wavelet transform-based features, Shukla et al. [11] used EDA-based features, and Tung et al. [16] extracted entropy-based features from EEG signals. Moreover, classification is a very important step in emotion recognition, and in the literature, it has been observed that different classifiers have been used for the detection of emotions [7,10,16]. Support vector machine (SVM) is the most commonly use classifier in emotion recognition [3,8,10,11] and it results in binary classification by giving an optimized hyperplane between the two classes. Convolutional neural network (CNN) is the most efficient classifier used for emotion recognition and it was used in many works, i.e., [17,18]. XGBoost model was used in [16] for emotion classification, while many other works [7,18,19] involved hybrid classifiers using SVM, CNN, Naïve Bayes, KNN, and LSTM [20,21,22,23,24]. In this study, we propose a novel method for the classification of physiological signals using stacked autoencoders. The major contributions of the proposed method are as follows: (1) an effective method for noise removal of physiological signals with a common cutoff frequency for multimodal signals. (2) A novel machine-learned feature extraction method for multimodal signals using custom stacked autoencoder architecture. (3) A lightweight and accurate ensemble classifier for the classification of emotions using multimodal signals. 

The rest of the paper is organized as: Section 2 discusses state-of-the-art techniques for emotion recognition using different physiological signals, Section 3 describes the proposed methodology, Section 4 performs an analysis of experimental results and Section 5 concludes the paper and presents future directions.

## 2. Literature Review

With the advancement in HCI technology in recent years, emotion recognition using physiological signals has gained significant attention in the research. Researchers used different training models and datasets for performing emotion recognition through physiological signals. Commonly used physiological signals are EEG, ECG, and GSR which serve the purpose of emotion recognition more efficiently. In recent research [7,18,19,20,21,22,23,24,25,26,27,28,29,30,31,32], researchers have proposed different emotion recognition methods using EEG, ECG, and GSR signals from different datasets including AMIGOS, DEAP, DREAMER, etc. The typical method for classification of these physiological signals includes three steps, i.e., pre-processing of physiological signals, features extraction, and classification. 

Noise added to physiological signals during the acquisition process may degrade the system’s performance; therefore, it is very important to clean the signals from all noise effects through pre-processing [8]. Cross-talk, measuring instruments, and other electromagnetic interferences make the physiological signals unsuitable for emotion recognition. Therefore, Sharma et al. [8] used the sliding mode singular spectrum (SM-SSA) method for decomposing EEG and ECG signals into reconstructed components (RCs), while Garg et al. [3] decomposed signals into equal-length samples using an approach of overlapping sliding windows (OSW) where he used 512 sized windows with a shift of 32. Window size and shift were calculated empirically. Raw physiological signals obtained from different participants and from different contexts may contain different types of artifacts, Klados et al. [21], Dar et al. [11], Tung et al. [16], and S. M et al. [32] performed down-sampling and bandpass filtering to remove the noise artifacts from signals, while Dar et al. [7], Zhao et al. [18] and Zhao et al. [14] further performed z-score normalization to remove baselines from signals to improve recognition accuracy. Sarkar et al. [15] proposed a self-supervised approach for ECG-based emotion recognition. Firstly, the former network was trained on pretext tasks using unlabeled data to learn spatiotemporal features and silent abstract representation of data. For the signal transformation recognition network, six transformations are performed for ECG signals.

After preprocessing of physiological signals, features are extracted from cleaned physiological signals for classification of signals into four classes (i.e., HAHV, HALV, LAHV, LALV). It has been observed that different researchers extracted different domains of hand-crafted features from preprocessed physiological signals [4,8,11,12,19,33,34,35,36,37,38,39,40]. Garg et al. [3] extracted two features, i.e., normalized wavelet energy (NWE) and band-power (NBP), and from the decomposed signals of EEG using Fourier and wavelet transform, respectively. He created a combined feature vector by appending five features of NBP and five features of NWE. Sharma et al. [8] extracted two different entropy-based features that were computed from RCs of EEG/ECG signals namely: information potential (IP) and centered correntropy (CEC). IP is invariant to the mean density of samples while CEC is the correlation that abstracts higher-order information of joint distribution. Granados et al. [12] and Shukla et al. [11] extracted different statistical features in the time domain, frequency domain, and non-linear domain. Statistical features including amplitude, time of decay, mean amplitude indices, rise time, and SCR peaks indices have also been computed for ECG signals [4]. Shukla et al. [11] extracted event-related features and statistical and Hjorth features in the time domain and many frequency domain features were extracted from five bands of EDA signals in the frequency domain. In time-frequency domain features such as discrete wavelet transform, stationary wavelet transforms features, Mel frequency cepstral coefficients (MFCC) and their statistical features were extracted. Tejada et al. [19] extracted different AMIGOS features from 14 channels of EEG signals. A total of 105 EEG features were used which were reported with the AMIGOS dataset and they referred to PSD and PSA features. Then, seven features were also utilized from age, sex, and five personality traits. Therefore, a total of 112 features were used from the AMIGOS dataset in this work. In total, 154 EEG features were added, and as a whole of total 266 features were used for the classification model.

There are few researchers who used automatedly generated features for emotion recognition [1,20]. Topic et al. [1] generated feature maps, i.e., TOPO-FM and HOLO-FM, and applied the convolutional layer separately on each characteristic feature, resulting in multiple feature matrices. These matrices were fused together in the form of a single feature matrix and were given as an input to machine learning-based classifiers for emotion classifications. Hu et al. [20] used a novel convolutional layer called the Scaling layer which could extract spectrogram-like features from raw EEG signals. This multi-kernel layer takes a 1D input signal and gives 2D spectrogram-like feature maps of a signal. Once the features are extracted, the next step is classification. Researchers have used machine and deep learning classifiers for the classification of four states of emotions based on valence and arousal. Machine learning methods include SVM, KNN, NB, DT, and MLP, whereas deep learning methods involve convolutional neural network variants and long-short-term memory units (LSTM). 

In recent studies, SVM was used for the classification of emotions in many works proposed by different researchers [1,8,11,19,29,30,31]. Sharma et al. [8] used the KNN classifier along with the SVM classifier, while Tejada et al. [19] used combinations of classifiers, i.e., SVM classifier, naïve Bayes, random forest, and artificial neural networks for the classification of emotions into different categories. However, deep learning methods, i.e., CNN, its customized versions, and DCNN have been proposed by multiple researchers [3,6,9,11,20]. Garg et al. [3] used 1D and 2D CNN architectures with max-pooling layers followed by four output dense layers. Granados et al. [12] used a deep convolutional neural network (DCNN) for classification. Feature vector extracted from physiological signals is fed to the input of fully connected layers (FCN) to train and validate the model. The last fully connected layer consists of output neurons for the prediction of the state. Rahim et al. [1] used the AlexNet architecture of CNN in their work where it has five convolutional layers and three fully connected layers. Automated features were extracted by different filters present in convolutional layers and there was a max pooling layer after the first and second convolutional layers and the third, fourth, and fifth convolutional layers were directly connected. Output was generated by the second fully connected layer and ReLU was applied after the last convolutional and fully connected layer. Hu et al. [20] introduced the ScalingNet, a network constructed by a series of scaling layers to perform emotion recognition using raw EEG signals. Sarkar et al. [17] used the CNN model for classification with 512 hidden layers in the architecture. Dar et al. [7] used two different architectures of DNN for classification through the neural network, one for EEG (2D CNN architecture) that would be used for the classification of images while the other one was for ECG and GSR signals which were built with the combination of LSTM and 1D convolutional network. 

It has been observed in the literature that most of the researchers have tested their emotion recognition methods on AMIGOS [1,3,7,8,11,12,21,38,39,40], DEAP [10,14,18,20], and DREAMER [7,8,10]. Furthermore, most commonly used physiological signals in the literature are EEG, ECG, and GSR [3,8,11,18,20,21,33,41]. These physiological signals have a complex and non-stationary nature. They are sensitive towards noise due to cross-talk, measuring instruments, and other electromagnetic interferences and therefore degrade the classification accuracy. Many preprocessing techniques such as bandpass filtering [11,16] and z-score normalization [14,18] have been proposed in the literature to address the issue of noise removal but still, there is significant room to improve this mechanism for improving recognition results. Moreover, many hand-crafted [4,8,11,12,19] and automated features [1,20] are discussed in the literature, but still, there is a need to have more discriminating features that could have strong relationships with the emotional changes in the human brain and human body when they are invoked with certain stimuli. Similarly, the selection of the classifier is a very important step in performing emotion recognition because the accurate results lead to the highest precision rate. Therefore, all these issues are addressed in our proposed work by using an independent component analysis (ICA) for preprocessing and stacked autoencoders for automated feature extraction. Moreover, the deep learning technique of LSTM is used in the proposed work for the final classification of emotions in a more efficient way. 

## 3. Methodology

**Figure 2:**
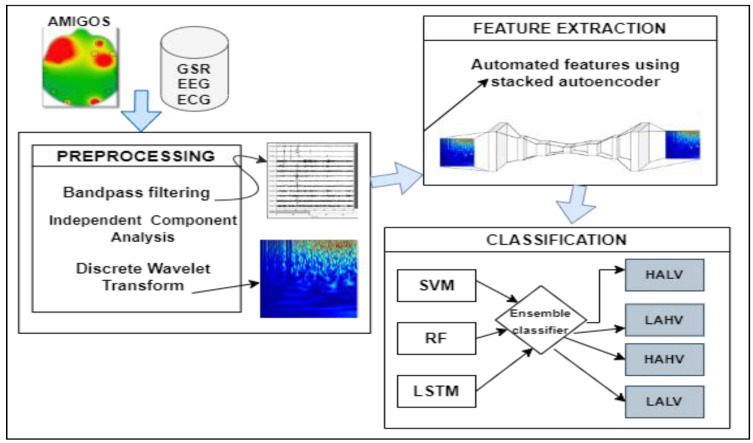
Block diagram of proposed method for emotion charting using multimodal signals.

We propose a method for the classification of physiological signals that classify emotions into four classes, i.e., HVHA, LVLA, HVLA, and LVHA. The flow diagram of proposed system is shown in Figure 2. The proposed system mainly consists of three steps: preprocessing, feature extraction, and classification. After performing different experiments with varying window sizes an overlapping window of 30 s with an overlap of 15 s has been selected to segment data into equal-sized segments. In preprocessing first bandpass filters are applied on physiological signals to remove power lines and baseline noise. After noise removal, ICA (independent component analysis) was applied to EEG signals followed by the discrete wavelet transform (DWT). In features extraction, stacked autoencoder is used to extract machine-learned features from the preprocessed signals and then SVM and LSTM were used to classify the emotions into four categories. Three main phases of the paper are discussed below:

### 3.1. Preprocessing of Physiological Signals

The complex and sensitive nature of physiological signals towards noise from cross-talk, measuring instruments, and other electromagnetic interferences invoke the need to perform preprocessing for the efficient physiological signals-based emotion recognition. Physiological signals have different types of noises including physiological artifacts and powerline and baseline interferences. These noises result in inefficient emotion recognition; therefore, it is important to eliminate these noises at the early stage of emotion recognition. For this purpose, first physiological signals EEG and ECG are filtered using bandpass filters (with cut-off frequency of 0.5 Hz to 45 Hz) to remove powerlines and baseline noise. Similarly, bandpass filter with ranges between 0.04 Hz to 0.25 Hz has been applied to GSR signals. After noise removal, independent component analysis (ICA) was applied only on 14 channels of EEG signals for further processing.

#### 3.1.1. Independent Component Analysis

Then, filtered EEG signals are sent to independent component analysis (ICA) which actually transforms the signal into a signal having mutually independent components [23]. Hence the independent components cannot deduce information from each other. Statistically, independence is computed by finding out the joint probability of the particular signal. Joint probability is computed by the product of probabilities of all the independent components.

Let us suppose we have m independent signals say $a_{i}\left(t\right)$ for ***i* = 1,…**, *m* where signal a is the function of *t* (1≤t≤T). Hence a(t) is the vector that has zero mean and is composed of m values. We further assume that signal a(t) has independent components and is noiseless signal, therefore we generate a function called multivariate density function using the probabilities of independent components which is written as

(1) $$p\left(a\left(t\right)\right)=\prod_{i=1}^{m}p\left(a_{i}\left(t\right)\right)$$

Let us suppose we have a d-dimensional data vector ***X*** which is observed at each moment,

(2) s(t)=Xa(t),

where ***X*** is m×d scalar matrix and d≥m. Independent component analysis actually needs to recover the source signal from the recognized signal. More precisely, we obtain a real matrix ***Y*** such that

***w(t) = Ys(t) = YXa(t)*** (3)

where ***w*** is the estimate of source signal ***a(t)***. Moreover, ***Y*** can be calculated using above equation as $Y=X^{-1}$, but both ***X*** and its inverse are unknown, and it could be found by using any of the determinant techniques of inverse. Then, estimate of source signal ***w(t)*** is forwarded to DWT for converting it into 2D signal. 

#### 3.1.2. Discrete Wavelet Transform for Multimodal Signals

Then, the output signal obtained from ICA is given to discrete wavelet transform (DWT). It is used to convert time domain to frequency domain signals. Discrete wavelet transform (DWT) implements orthonormal wavelet transform in discrete time context [24]. In DWT mother wavelet determines the decomposition of wavelets which consists of consequent low-pass and high-pass filtering. Wavelet function has two properties, i.e., scaling and translating which are represented in the equation given below:

(4) $$\emptyset_{j,k}\left(t\right)=2^{\frac{j}{2}}\emptyset \left(2^{j}t-k\right),$$

(5) $$\psi_{j,k}\left(t\right)=2^{\frac{j}{2}}\psi \left(2^{j}t-k\right),$$

where *j* represents dilation and *k* represents position. General equation of dilation is shown below:

(6) $$\phi \left(t\right)=\sum_{m}l_{\phi }\left[m\right]\sqrt{2}\phi \left(2t-m\right),$$

where $l_{\phi }\left[n\right]$ is the discrete low pass filter.

The relationship of wavelet function ψ(t) and ϕ(t) is shown below:

(7) $$\psi \left(t\right)=\phi \left(t\right)=\sum_{m}l_{\psi }\left[m\right]\sqrt{2}\phi \left(2t-m\right)$$

where relationship with wavelet coefficient and low pass filter is shown in given equation.

(8) $$l_{\psi }\left[m\right]={\left(-1\right)}^{m}l_{\phi }\left[1-m\right]$$

The wavelet transform is then forwarded to autoencoder for features extraction. 

### 3.2. Feature Extraction from Multimodal Signals

Features extraction is a very important phase in emotions recognition method. Features are extracted through autoencoder in our proposed method. 

#### Customized Stacked Autoencoder

**Figure 3:**
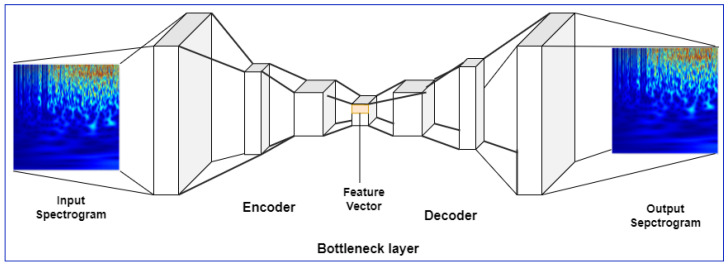
Visualization of the proposed architecture of stacked autoencoder.

Autoencoder works on the basis of back propagation mechanism which is used to learn low dimensional data into high dimensional data by using the significant information from input data [25,26]. Generally, the model is built by minimizing the difference between input and output; hence, the middle layer represents the compressed form of input [27]. Its architecture has three parts: input layers, hidden layers, and output layers which are shown in Figure 3. Input and output layers have same dimensions so the network from input to hidden layer is called “encoder network” (EN) and hidden layer to output layer is called “decoder network”. 

In our proposed system the encoder network consists of set of 3 convolutional blocks which take wavelet transform of EEG, ECG, and GSR signals as input. Each convolutional block consists of convolutional layer with 15 × 15, 9 × 9, and 3 × 3 sized kernels, respectively, for each block. After convolutional layer we have Batch normalization layer followed by ReLU layer, max pooling layer, and dropout layer. The hidden layer which is also called “bottleneck” layer contains the compressed output with its important features. Finally, decoder network consists of up-sampling and convolutional blocks that reconstruct the output of bottleneck. The convolutional blocks consist of transpose convolutional layer, batch normalization, ReLU, and max-unpooling layers followed by dropout layer. The transpose convolutional layer works exactly as convolutional layer but in a reverse manner, hence increasing the width and height of input layers. 

Autoencoder is trained according to the rule that minimizes the reconstruction loss function between actual data and rebuilt data (wavelet). We have to make sure that the derivative of the bottleneck activations is small than the input layer while training the model. Mathematically,

(9) $$derivative\;of\;bottleneck\;layer\;and\;input\;layer=\frac{\delta b}{\delta m}$$

where ***b*** is the bottleneck (hidden layer) and ***m*** is the input layer. 

However, the loss function could be represented as:

(10) $$L-\left|m-m^{2}\right|=\lambda \sum_{i}\|\Delta_{m}a^{b}\left(m\right){\|}^{2}$$

where *b* is the bottleneck or hidden layer for which the gradient is calculated and represented with respect to the input m as $\Delta_{m}a^{b}\left(m\right).$ Once the autoencoder is trained, the feature vector is obtained from the bottleneck layer of stacked autoencoder which is given to the classifiers as input for emotions classification. 

### 3.3. Classification

An ensemble classifier based on the majority voting of the three classifiers including LSTM with 32 repeating units, Random Forest (RF), and SVM with linear kernel function has been applied. RF combines the decision trees, whereas SVM draws a hyperplane for decision boundary. LSTM works very much like RNN at a very high level [28,41]. It consists of three parts which are known as gates of LSTM namely forget gate, input gate, and output gate. 

Forget gate: in the first gate of LSTM network, it checks the relevancy of input data and decides whether we should retain our information or forget it from the previous timestamp. Here is the equation for forget gate:

(11) $$y_{t}=\sigma \left(a_{t}*A_{f}+h_{t-1}*H_{f}\right),$$

where

$a_{t}$: input to the current timestamp

$A_{f}$: input weight matrix 

$h_{t-1}$: hidden state of the previous timestamp

$H_{f}$: weight matrix associated with hidden state

Later, sigmoid function is applied over the function $y_{t}$ to make the output in 0 and 1. This $y_{t}$ is then multiplied with previous cell state which are as shown:

(12) $$C_{t-1}*y_{t}=0,if\;y_{t}=0\;(\mathrm{forget}\;\mathrm{everything})$$

(13) $$C_{t-1}*y_{t}=C_{t-1},if\;y_{t}=1\;(\mathrm{forget}\;\mathrm{nothing})$$

If $y_{t}=0$ then the network will forget everything, if $y_{t}=1$, then the network will forget nothing.

Input gate: input gate quantifies important information carried by the input. Here is the equation of input gate:

(14) $$I_{t}=\sigma \left(a_{t}*A_{i}+h_{t-1}*H_{i}\right),$$

where

$a_{t}$: input in the current timestamp

$A_{i}$: weight matrix of input

$h_{t-1}$: represents hidden state at time ***t* − 1**

$H_{f}$: hidden state weight matrix

The function $I_{t}$ is passed through sigmoid function so it will result in either 0 or 1. 

Now new information is expressed as:

(15) $$n_{t}=tanh\left(a_{t}*U_{c}+h_{t-1}*H_{c}\right),\;(\mathrm{new}\;\mathrm{information})$$

New information needs to be passed to next state is the function of input state at current timestamp and function of hidden state at previous time stamp. It is the function of tanh which could be either −1 or 1. If the value of function $n_{t}$ is negative the information will be deducted from the cell state and if $n_{t}$ is positive the information will be added to the cell state. 

The equation used to add $n_{t}$ in the cell state is shown below:

(16) $$c_{t}=y_{t}*c_{t-1}+I_{t}*n_{t},\;(\mathrm{updating}\;\mathrm{cell}\;\mathrm{state})$$

$c_{t-1}$ is the cell state at current timestamp while rest of the values are calculated previously. 

Output gate: the output gate is equated as:

(17) $$o_{t}=\sigma \left(a_{t}*A_{o}+h_{t-1}*H_{o}\right)$$

As the activation function used is sigmoid function, so $o_{t}$ will result either 0 or 1. 

The current hidden state is the function of long-term memory ($c_{t}$) and current output ($o_{t}$) and it could be calculated

(18) $$h_{t}=o_{t}*tanh\left(c_{t}\right),$$

Feature vector generated from the previous step is fed to classifiers SVM, LSTM and RF. Then, single output is generated by using the approach of majority voting which constitutes the ensemble classifier.

## 4. Results and Discussion

We have trained the proposed system on the AMIGOS dataset which consists of ECG, EEG, and GSR signals from 40 participants which were recorded in two experimental settings. *k*-fold cross-validation has been used with *k* = 10 and each subject has multiple samples. Experiments were repeated for 10-fold validation and the average of all folds has been reported. Therefore, in every fold test and train, data are different in each fold. We performed multiple experiments to carry out the systematic evaluation of our proposed system using *k*-fold cross-validation for splitting samples of all classes into train and test with 2000 samples in the test. The performance measures used for the assessment of proposed system are accuracy, specificity, and sensitivity. Accuracy is calculated for each experiment; however, specificity and sensitivity are calculated for each label, i.e., (LAHV, HALV, LALV, and HAHV). The values of these performance measures are calculated using the given equations [18]:

(19) $$Accuracy=\frac{T_{P}+T_{N}}{Total\;numbers\;of\;samples}$$

(20) $$Specificity=\frac{T_{N}}{T_{N}+F_{P}}$$

(21) $$Sensitivity=\frac{T_{P}}{T_{P}+F_{N}}$$

$T_{P}-True\;positive$

$T_{N}-True\;negative$

$F_{P}-False\;positive$

$F_{N}-False\;negative$

In our system, we have two main categories, i.e., arousal and valence, which are further divided into four categories (i.e., HALV, HAHV, LALV, LAHV). Let us suppose arousal is true class and valence is false class. Yet for four classes one vs. all approach is used where three classes will become one class and rest will be another class. Therefore, true positive is when system predicts the true class accurately and true negative is when system predicts false class accurately. Similarly, a false positive is when system predicts a true class inaccurately and a false negative is when system predicts a false class inaccurately. 

Table 1 shows the results obtained with different experiments. First of all, the system was assessed without performing preprocessing and CNN was used to classify emotions into four classes, i.e., LAHV, HALV, HAHV, and LALV. In this experiment, with no preprocessing, an accuracy of 81.5% was observed with an average specificity of 91%. In the second experiment system, preprocessing was performed using a bandpass filter. It has been observed that the system’s accuracy has been improved by doing preprocessing and it is equal to 84%. Therefore, it is important to remove signals baseline and other types of noise for emotion recognition. In the third experiment, preprocessing is further enhanced by using Independent component analysis (ICA) which removes powerlines, etc., and improves the system’s overall performance.

In this experiment, system obtained an accuracy of 88.5%, which is a significant improvement as compared to the conventional preprocessing. In fourth experiment, after preprocessing features are extracted using autoencoder which gives a feature vector in its bottleneck layer. The feature vector is then forwarded to fully connected layer of CNN. It has been observed system achieved an accuracy of 89% and specificity of 94%. In fifth experiment, stacked autoencoder was used for feature extraction which consists of multiple CNN layers and maxpooling layer in its encoder network. Therefore, feature vector obtained from its bottleneck layer is then forwarded to FC layer of CNN. It has been observed in this experimentation that system’s performance gets improved by 1–2% in terms of accuracy. In sixth experiment, CNN classifier has been replaced by random forest (RF) which increases system’s accuracy which is equal to 90.5%. However, in the next experiment, classification was performed by an SVM classifier which increases system’s accuracy significantly by 2–3% and is equal to 92.5%. In seventh experiment, LSTM was used to classify emotions. System’s accuracy is further increased by using LSTM as compared to other classifiers which are equal to 93.5%. Finally, ensemble classifier is used in last experiment which takes the outputs of SVM, RF, and LSTM and results the best accuracy out of the three outputs. Overall system’s performance is improved by 1% with ensemble classifier. 

**Figure 4:**
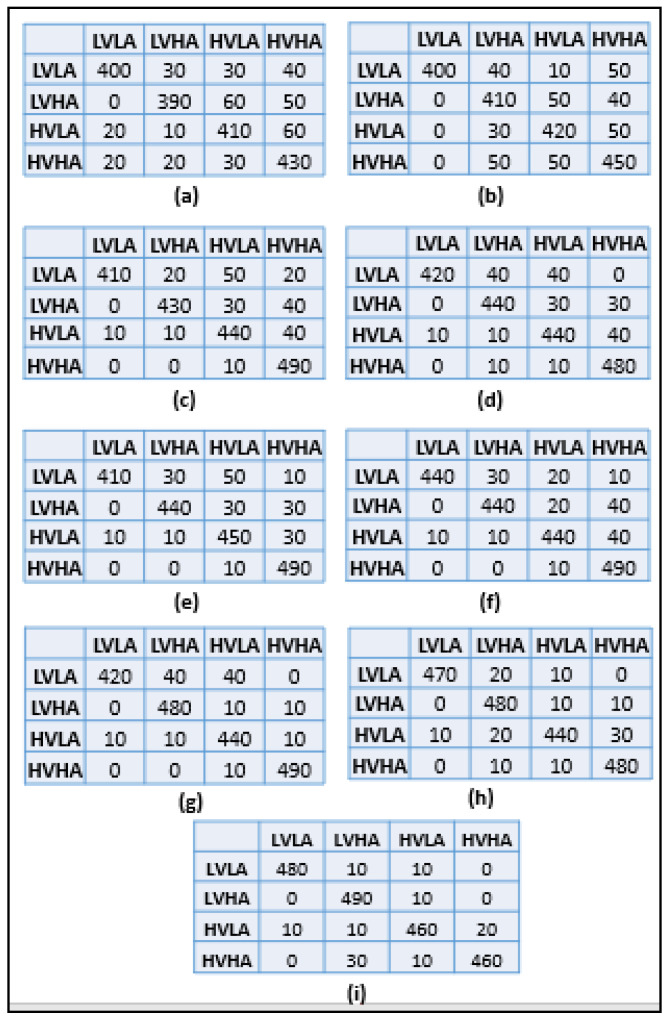
(**a**) Confusion Matrix for Experiment 1 (No preprocessing, DWT, CNN), (**b**) Confusion Matrix for Experiment 2 (Bandpass filtering, DWT, CNN), (**c**) Confusion Matrix for Experiment 3 (Bandpass filtering, ICA, DWT, CNN), (**d**) Confusion Matrix for Experiment 4 (Bandpass filtering, ICA, DWT, Autoencoder, CNN), (**e**) Confusion Matrix for Experiment 5 (Bandpass filtering, ICA, DWT, Stacked Autoencoder, CNN), (**f**) Confusion Matrix for Experiment 6 (Bandpass filtering, ICA, DWT, Stacked Autoencoder, RF), (**g**) Confusion Matrix for Experiment 7 (Bandpass filtering, ICA, DWT, Stacked Autoencoder, SVM), (**h**) Confusion Matrix for Experiment 8 (Bandpass filtering, ICA, DWT, Stacked Autoencoder, LSTM), (**i**) Confusion Matrix for Experiment 9 (Bandpass filtering, ICA, DWT, Stacked Autoencoder, Ensemble Classifier).

Figure 4 shows confusion matrix for all experiments where diagonal values in each matrix are correctly classified samples and off-diagonal values are incorrectly classified samples.

**Figure 5:**
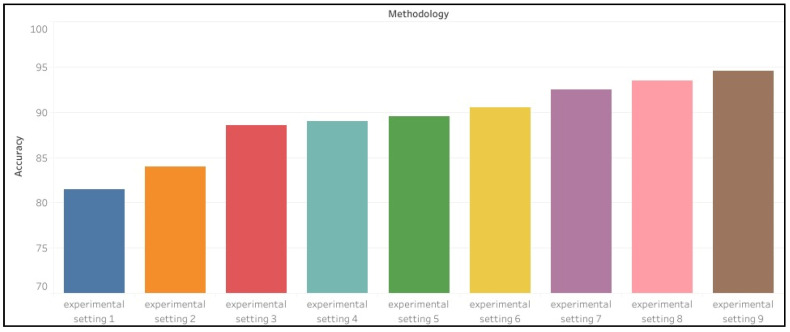
Performance of different proposed experimental settings.

**Figure 6:**
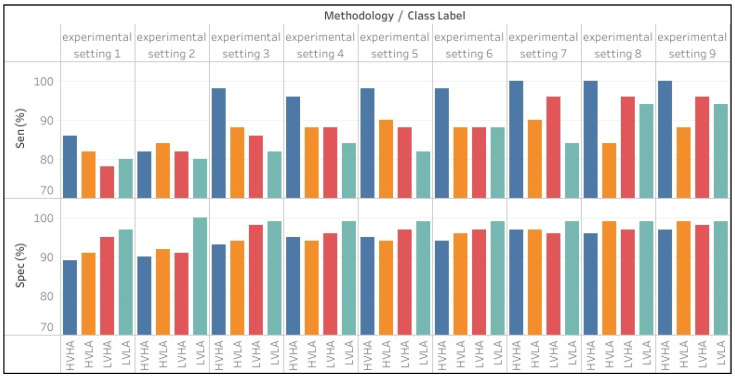
Sensitivity and specificity of different experimental settings.

Figure 5 shows performance of proposed experimental settings in terms of accuracy. It can be clearly seen that system performs better with ensemble classifier. Figure 6 shows overview of sensitivity and specificity of proposed experimental settings against each class label. 

**Figure 7:**
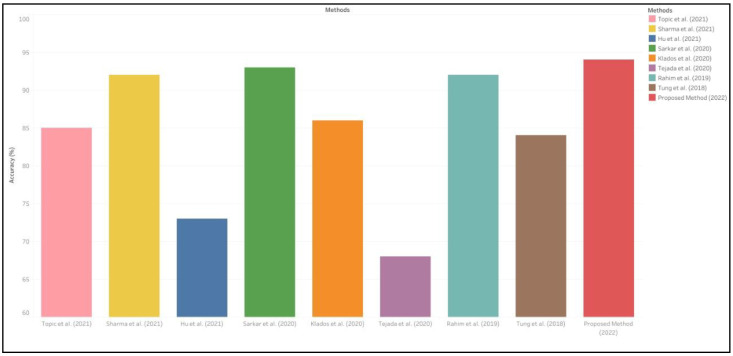
Comparison of proposed method with previous state-of-the-art method [1,8,10,16,17,19,20,21].

Table 2 shows the comparative analysis of the proposed method with previous state-of-the-art methods on emotion recognition. It is clearly observed in the table that methods with preprocessing give improved results as compared to methods with no preprocessing. Moreover, proposed method achieved the highest accuracy of 94.5% as compared to other state-of-art methods on emotion recognition which is also clearly seen in bar charts shown in Figure 7. 

## 5. Conclusions and Future Work

Emotion recognition is one of the most captivating topics in recent research. Many researchers came up with emotion recognition as discussed in the literature, but physiological signals-based methods are rarely implemented specifically with EEG, ECG, and GSR signals. Moreover, it has been found in the literature that many researchers have not addressed preprocessing and feature extraction phases efficiently. As the physiological signals are non-stationary and have powerline and baseline noise, this needs to be addressed properly. Moreover, there was a need to have the most discriminating features, which could draw the relation between human body signals and emotions. Therefore, this paper addressed these issues and proposed a method in which preprocess signals with independent component analysis and features are extracted using a stacked autoencoder using their bottleneck layer. For the classification of emotions into four categories (HALV, LAHV, LALV, HAHV), majority voting has been applied to the outputs of the three classifiers including SVM, RF, and LSTM. The system achieved the highest accuracy of 94.5% which outperforms the previous methods. There are certain limitations in our work as well, for instance, analysis of frequency bands has not been completed for the physiological signals and more deep learning models could be used for emotion recognition. This can be extended in the future to overcome the aforementioned limitations. Moreover, in the future, this work could be a great step towards the invention of wearable devices which will help in assessing the emotions of the person suffering from depression and other brain disorders. Moreover, these devices can be installed in hospitals as well which can assist doctors in the treatment of patients with depression and autism. Cut-off frequencies for bandpass filtering for EEG, ECG, and GSR signals can be varied to observe the changes in the emotions for improved classification. Real-time data acquisition could be completed in the future and the proposed system could be tested on real-time datasets as well, which would add more excellency to the proposed work.

## Figures and Tables

**Table 1:** Results obtained on AMIGOS dataset by varying different experimental setups.

Methodology	Accuracy (%)	Sensitivity (%)	Specificity (%)
No preprocessing DWT CNN	81.5	80	97
		78	95
		82	91
		86	89
Bandpass filtering DWT CNN	84	80	100
		82	91
		84	92
		82	90
Bandpass filtering ICA DWT, CNN	88.5	82	99
		86	98
		88	94
		98	93
Bandpass filtering ICA DWT Autoencoder CNN	89	84	99
		88	96
		88	94
		96	95
Bandpass filtering ICA DWT Stacked Autoencoder, CNN	89.5	82	99
		88	97
		90	94
		98	95
Bandpass filtering ICA DWT Stacked Autoencoder RF	90.5	88	99
		88	97
		88	96
		98	94
Bandpass filtering ICA DWT Stacked Autoencoder SVM	92.5	84	99
		96	96
		90	97
		100	97
Bandpass filtering ICA DWT Stacked Autoencoder LSTM	93.5	94	99
		96	97
		84	99
		100	96
Bandpass filtering ICA DWT Stacked Autoencoder Ensemble Classifier	94.5	94	99
		96	98
		88	99
		100	97

**Table 2:** Comparison of proposed approach with existing methods.

Method	Year	Dataset	Preprocessing	Feature Extraction	Classifier	Accuracy (%)
Topic et al. [10]	2021	DEAP SEED DREAMERS AMIGOS	--	TOPO-FM, HOLO-FM, deep learning features extractor (CNN)	SVM	85.07%
Sharma et al. [8]	2021	DREAMER AMIGOS	Decomposition of signals into reconstructed components (RCs)	Entropy based features: Information potential (ip) and centered correntropy (CEC)	KNN SVM	92.38%
Hu et al. [20]	2021	DEAP AMIGOS	--	Spectrograms such as feature maps	Scaling net neural network	73.77%
Sarkar et al. [17]	2020	AMIGOS DREAMER WESAD SWELL	Down sampling, high pass IIR filter and z-score normalization	--	SVM, Fully supervised CNN, KNN, RF, LDA	93.8%
Klados et al. [21]	2020	AMIGOS	--	Cross-spectrum, coherence, betweenness centrality (BC),	SVM	86.5%
Tejada et al. [19]	2020	AMIGOS	Bandpass filter	PSD, PSA, fractional dimension (FD), differential entropy (DE), rational asymmetry (RASM), differential asymmetry (DASM)	SVM, Naïve Bayes, RF, ANN	68%
Rahim et al. [1]	2019	AMIGOS	SMOTE technique	Scalogram, spectrogram	CNN	92.70%
Tung et al. [16]	2018	AMIGOS	Noise removal using different filters	ANOVA statistical analysis	XGBoost model	84%
**Proposed Method**	**2022**	**AMIGOS**	**Bandpass filter, Independent Component Analysis (ICA), DWT**	**Stacked Autoencoder**	**Ensemble Classifier**	**94.5%**

## Data Availability

The data used in this research can be obtained from the corresponding authors upon request.
